# Mast Cells in Liver Fibrogenesis

**DOI:** 10.3390/cells8111429

**Published:** 2019-11-13

**Authors:** Ralf Weiskirchen, Steffen K. Meurer, Christian Liedtke, Michael Huber

**Affiliations:** 1 Institute of Molecular Pathobiochemistry, Experimental Gene Therapy and Clinical Chemistry (IFMPEGKC), University Hospital, RWTH Aachen University, D-52074 Aachen, Germany; smeurer@ukaachen.de; 2 Department of Internal Medicine III, University Hospital, RWTH Aachen University, D-52074 Aachen, Germany; cliedtke@ukaachen.de; 3 Institute of Biochemistry and Molecular Immunology, Medical Faculty, RWTH Aachen University, D-52074 Aachen, Germany

**Keywords:** liver, inflammation, fibrosis, mast cell, degranulation, TGF-β, animal models, translational medicine, chymase, tryptase

## Abstract

Mast cells (MCs) are immune cells of the myeloid lineage that are present in the connective tissue throughout the body and in mucosa tissue. They originate from hematopoietic stem cells in the bone marrow and circulate as MC progenitors in the blood. After migration to various tissues, they differentiate into their mature form, which is characterized by a phenotype containing large granules enriched in a variety of bioactive compounds, including histamine and heparin. These cells can be activated in a receptor-dependent and -independent manner. Particularly, the activation of the high-affinity immunoglobulin E (IgE) receptor, also known as FcεRI, that is expressed on the surface of MCs provoke specific signaling cascades that leads to intracellular calcium influx, activation of different transcription factors, degranulation, and cytokine production. Therefore, MCs modulate many aspects in physiological and pathological conditions, including wound healing, defense against pathogens, immune tolerance, allergy, anaphylaxis, autoimmune defects, inflammation, and infectious and other disorders. In the liver, MCs are mainly associated with connective tissue located in the surrounding of the hepatic arteries, veins, and bile ducts. Recent work has demonstrated a significant increase in MC number during hepatic injury, suggesting an important role of these cells in liver disease and progression. In the present review, we summarize aspects of MC function and mediators in experimental liver injury, their interaction with other hepatic cell types, and their contribution to the pathogenesis of fibrosis.

## 1. Introduction

### 1.1. Mast Cell Development

Mast cells (MCs) are hematopoietic cells of the myeloid lineage [1]. They can be found particularly in tissues with close contact to the environment, such as skin, gastrointestinal tract, upper airways, and lung [2]. However, MCs are also located in other vascularized organs (e.g., liver and kidney [3,4]). Correlating with their presence in various locations, MCs present as a highly heterogeneous cell population with subtype-dependent differences in cell morphology, histochemical properties, expression of granular proteases, and function, amongst others [5]. This intriguing plasticity and heterogeneity also have their origin in the differentiation process of MCs [6].

MCs originate from hematopoietic stem cells, which differentiate into MC precursors (MCps) in the bone marrow. MCps then leave the bone marrow and distribute via the blood and transendothelial migration into target tissues, where they eventually phenotypically mature in the presence of tissue-specific factors, such as cytokines, growth factors, and extracellular matrix (ECM) components. Different maturation conditions lead to functionally diverse MCs, which can be subdivided in mice in chymase-expressing mucosa-type MCs (MMCs) and tryptase/chymase-expressing connective tissue-type MCs (CTMCs).

From a signaling perspective, MC development in vivo is strictly dependent on the expression of the receptor tyrosine kinase KIT, also known as CD117 [7]. Likewise, expression of the KIT ligand stem cell factor (SCF) is mandatory for MC development in vivo [8]. Another prominent MC receptor is the high-affinity receptor for IgE (FcεRI) [9], which is important, amongst other factors, for defense against helminths, but also for the induction of allergic reactions. The co-expression of both KIT and FcεRI, in addition to distinct cytoplasmic metachromatic granules (also termed secretory lysosomes), characterizes mature MCs [2].

With respect to the distribution of MCps through the blood to respective target tissues, productive interactions between surface molecules of MCps and endothelial cells are required for transendothelial migration [10]. As an example, the homing of MCps to the small intestine is dependent on the α4β7 integrin on the MCps, and MAdCAM-1 and VCAM1 as counterligands on the endothelial cells [11]. For α4β7 integrin activation by inside-out signaling, ligand-induced activation of CXCR2 on MCps is required [12]. After migration to the different tissues, MCps differentiate into tissue-specific mature MCs, which can belong to either of the two major subclasses of MCs, MMCs or CTMCs. In addition to the subtype- and tissue-specific changes in secretory potential (e.g., differential content of secretory lysosomes and configuration of signaling systems), mature MCs downregulate cell-surface adhesion molecules and chemokine receptors [13,14]. Intriguingly, by using a new fate mapping mouse model, Gentek et al. recently demonstrated that MCs have dual developmental origins [15]. Whereas most skin MCs are of primitive origin during embryogenesis (i.e., yolk sac derived), adult definitive MCs originate from definitive hematopoietic stem cells of the aorta-gonad-mesonephros vascular endothelium. Moreover, in this study it was demonstrated that replenishment of adult tissue MCs predominantly occurs via the proliferation/differentiation of long-lived tissue-resident precursors [15]. These data were in principle verified by Li et al. [16]. However, by applying two different fate mapping mouse models, they could demonstrate that most CTMCs even derive from late erythro-myeloid progenitors generated at the hemogenic endothelium of the yolk sac.

### 1.2. Different Roles of Mast Cells in Physiology and Pathophysiology

MCs take part in processes of innate and adaptive immunity, alerting the body to invasion by bacteria and parasites, and regulating lymphocyte reactions [17,18]. Moreover, through the release of different proteases, MCs are able to attenuate snake-venom-induced and honeybee-venom-induced pathology by degrading the proteinaceous venom components [19]. They also promote homeostasis by limiting endothelin-1-induced toxicity in a carboxypeptidase-A3-dependent manner [20,21]. Also, on the beneficial side, MCs have a positive impact on bone repair, most likely by recruiting vascular endothelial cells during the inflammatory phase, and by coordinating anabolic and catabolic activities during tissue remodeling [22]. Moreover, myocardial MCs regulate heart function after a myocardial infarction [23], and mucosal MCs in the intestinal epithelium execute anti-helminth immunity [24].

In addition to these beneficial functions, MCs play detrimental roles as well, e.g., as central effector cells in acute allergic disorders (such as rhinitis, asthma, and anaphylactic reactions) [25]. Moreover, MCs promote T cell-driven collagen-induced arthritis. However, they are dispensable for antibody-induced arthritis in which T cells are bypassed [26]. MCs are also known to modulate the growth/metastasis of certain solid tumors [27] and can contribute to the development of various fibrotic diseases [28]. In septic peritonitis, MCs have been demonstrated to suppress the phagocytosis of bacteria by peritoneal macrophages in an IL-4-dependent manner. Thus, MCs can aggravate the outcome of severe bacterial infections [29]. Neither the number of positive nor the number of negative functions of MCs mentioned above are exhaustive; nevertheless, they provide an idea about the variety of the biological functions of MCs.

In addition, detrimental outcomes are known for patients suffering from mastocytosis, which is a rare and heterogeneous disease characterized by the expansion and accumulation of clonal (neoplastic) tissue MCs in one or more organ systems [30,31]. Mastocytosis can be divided into subvariants of cutaneous mastocytosis, different types of systemic mastocytosis (SM), and localized MC tumors. SM is further divided into several subtypes (i.e., indolent SM, smoldering SM, SM with an associated hematologic neoplasm, aggressive SM, and MC leukemia [30,31]). The different subtypes of mastocytosis exert highly variable clinical courses, ranging from asymptomatic with a normal life expectancy to fatal with high mortality within months or weeks [30,31]. Additionally, clinical conditions related to MC activation, where symptoms are recurrent, are accompanied by an increase in MC-derived mediators in biological fluids, and are responsive to treatment with MC-stabilizing or mediator-targeting drugs, can/might be diagnosed as MC activation syndrome (MCAS) [32] (for a proposed diagnostic algorithm for MCAS we refer to Valent et al. [33]). In conclusion, MCs can be regarded as a versatile cell type with differential functions in physiological and pathophysiological settings, and hence, MCs are very attractive drug targets.

### 1.3. Mast Cell Mediators

The most eye-catching feature of MCs visualized using electron microscopy is the multitude of electron-dense vesicles/granula. These organelles are secretory lysosomes that store preformed pro-inflammatory mediators (e.g., histamine; proteoglycans, such as heparin and chondroitin-sulfate; various proteases, such as tryptase, chymase, carboxypeptidase A3, granzyme B, and active caspase-3; and certain cytokines) that are released immediately upon antigen-triggered activation of the IgE-bound FcεRI in a process called degranulation [34,35,36,37]. Another important MC-selective receptor able to induce the process of degranulation upon recognition of its ligand(s) is the Mas-related G protein-coupled receptor X2 (MRGPRX2; in mice: MRGPRB2), which can trigger the secretory response upon binding to antimicrobial peptides, neuropeptides, eosinophil peroxidase, and various peptidergic drugs, amongst others [38]. Before the identification of this receptor, many detrimental reactions in patients were referred to as “drug-induced pseudoallergies” without having the notion that it is only one receptor that recognizes this variety of different molecules [39].

A further immediate response of MCs is the generation and release of arachidonic acid metabolites, particularly the leukotrienes LTB_4_ and LTC_4_, and prostaglandin PGD_2_. These mediators, in a situation-dependent context, are able to initiate, amplify, or attenuate inflammatory responses. Furthermore, they can influence the magnitude, duration, and nature of subsequent immune responses [40]. In addition, MCs are capable of producing and secreting numerous cytokines, chemokines, growth factors, and angiogenic factors mediating multiple pro-inflammatory, anti-inflammatory, and/or immunoregulatory effects in a situation-dependent manner [2,41]. Amongst others, MCs are able to synthesize and secrete TNF-α, IL-1β, IL-3, IL-5, IL-6, IL-8, IL-9, IL-13, CCL5, TGF-β1, and FGF.

The composition of MC responses is largely dependent on the activated receptor(s). In response to an antigen, MCs degranulate and produce arachidonic acid metabolites, as well as cytokines and chemokines. In contrast, the stimulation of MCs via cytokines or pattern recognition receptors (e.g., toll-like receptors (TLRs)) only induces the generation of arachidonic acid metabolites and cytokines/chemokines. With respect to TLR4 activation by lipopolysaccharide (LPS), MCs are markedly different compared to macrophages concerning their receptor composition and organization of signaling pathways. MCs do not express the GPI-anchored protein mCD14, which affects the chemotype of recognized LPS molecules (R-LPS >> S-LPS) [42]. Moreover, upon LPS recognition, MCs do not activate the TRIF pathway, and thus, MCs do not produce IFN-β [43,44].

### 1.4. Murine Models to Study Mast Cell Involvement

To study the role of MCs in different disease situations, mostly mouse models are used, though models in rats, hamsters, dogs, and rabbits have also been used for the investigation of certain diseases. Most mouse studies have made use of animals that do not express KIT and thus are devoid, though not completely, of MCs (“*Kit* mutant MC-deficient mice”). Different mutant mice carrying mutations in the *Kit* gene/locus have been frequently used (e.g., WBB6F_1_-*Kit^W/W-v^* and C57BL/6-*Kit^W-sh/W-sh^* mice [7,45]) to study disease development in the absence of MCs. Moreover, in vitro differentiated bone-marrow-derived MCs (BMMCs) have been used to engraft an MC population in these genetically MC-deficient mice (“MC knock-in mice”) and disease development has been studied [46]. If changes in MC-deficient mice, compared to the respective wild-type mice, could be reverted via the re-establishment of MC populations, then this was taken as a proof of MC involvement in the particular disease process. However, it should be noted that KIT is also expressed on hematopoietic stem cells and almost all myeloid progenitor cells, allowing for altered adaptive and innate immune reactions in KIT-deficient mice, which cannot only be attributed to missing MCs. Not unexpected, using a *Kit*-independent mouse model of MC deficiency (Cpa3^Cre/+^ mice (Cre-Master mice) carrying a targeted insertion of Cre recombinase in the carboxypeptidase A3 locus), Feyerabend et al. could not verify all results from studies that used *Kit*-dependent MC-deficient mouse models [47]. Examples of such MC-independent diseases were antibody-induced autoimmune arthritis and experimental autoimmune encephalomyelitis. Additional mutant mice with constitutive MC deficiency unrelated to *Kit* abnormalities are the *Mcpt5-Cre*; *R-DTA* mice and the *Cpa3-Cre*; *Mcl1^fl/fl^* mice. For the generation of *Mcpt5-Cre*; *R-DTA* mice, *Mcpt5-Cre* transgenic mice [48] were crossed with *R-DTA^fl/fl^* mice [49] to yield a mouse strain in which CTMCs are ablated by the expression of the diphtheria toxin α chain [50]. For the generation of transgenic *Cpa3-Cre*; *Mcl1^fl/fl^* mice (also known as “Hello Kitty” mice), mice expressing *Cre* under the control of a *Cpa3* promoter fragment were crossed with *Mcl1^fl/fl^* mice [51], allowing for the deletion of the gene of the anti-apoptotic factor MCL1 [52]. The different mouse models of MC deficiency have been comprehensively reviewed by Galli et al. [53].

## 2. Fibrosis: Some General Aspects

The term fibrosis describes a pathological situation defined by the overgrowth, hardening, and excessive scarring that can affect nearly all tissues [54]. The scarring process is mainly characterized by the replacement of normal parenchymal tissue by connective tissue. The process is initiated by neutrophilic inflammation, which can result from various stimuli, such as mechanical injury, infections, autoimmune attacks, toxins, or radiation. Mechanistically, this process aims to eliminate the initial cause of injury and preserve the function of the affected organ [55].

The primary inflammatory response is well-orchestrated and requires engagement of the local vascular system and components of the immune system, as well as the systemic coordination of endocrine and neurological mediators [54]. This interconnection is driven by a variety of soluble factors (chemokines, cytokines). During acute inflammation, resident immune cells (e.g., macrophages, dendritic cells, MCs) are the most important in the initial phase. These cells are equipped with pattern recognition receptors (PRRs) playing a crucial role in the detection of pathogen-associated molecular patterns (PAMPs) and damage-associated molecular patterns (DAMPs) [54,55]. These receptors differ in their ligand recognition and defined subsets can identify a broad range of proteins, nucleic acids, or glycans [56,57]. After ligand recognition, these receptors induce various cellular responses resulting in the release of different inflammatory mediators that in turn provoke the typical five cardinal clinical signs of inflammation, namely *rubor* (redness), *calor* (heat), *tumor* (swelling), *dolor* (pain), and *functio laesa* (loss of function).

If this first-line defense is insufficient to eliminate the disease-causing agent and inflammation persists, various immune cells, such as macrophages and T-lymphocytes, are triggered to produce high quantities of cytokines and enzymes, which subsequently provoke more lasting damage. As a consequence, parenchymal cell death occurs, which is associated with an uncontrolled release of pro-fibrogenic mediators that in turn lead to activation of a pro-fibrogenic cell population with the capacity to synthesize large quantities of ECM components [58]. In this regard, members of the TGF-β family of cytokines are of fundamental importance, acting as a common master switch. TGF-β strongly promotes the synthesis of collagen and fibronectin in both epithelial and mesenchymal cells and further suppresses the process of inflammation [58]. Other important soluble mediators triggering the process of fibrogenesis are members of the platelet-derived growth factor (PDGF) family and connective tissue growth factor (CTGF). While PDGFs are highly competent mitogens and chemoattractants for fibrogenic cells, CTGF contains structural features serving as TGF-β binding domains, thereby enhancing its biological activity or sequestering other members of the TGF-β gene family, such as bone-morphogenetic proteins (BMPs), which usually act as opposing factors for TGF-β [59]. In concert with TGF-β, these mediators increase the number and activity of myofibroblasts (MFBs) and their progenitors, thereby promoting fibrogenesis in a variety of organs including skin, heart, kidney, pancreas, lung, liver, and others. Strikingly, the population of cells capable of synthesizing ECM consists of resident pro-fibrogenic cells, such as hepatic stellate cells (HSCs) and portal myofibroblasts, progenitors invading the inflamed tissue, and cells that become activated and acquire fibrogenic features [55]. These cells are preserved between different organs. The final ECM-producing cell type, the myofibroblast, can originate from cellular subsets including resident fibroblasts, mesothelial cells, circulating fibrocytes, epithelial cells, endothelial cells, pericytes, vascular smooth muscle cells, Gli1^+^ perivascular mesenchymal stem cell-like cells, and other more specialized cells that are present within different organs or tissue. In kidney, these are, for example, podocytes found in the lining of the Bowman’s capsules in the nephrons or tubular epithelial cells that might also acquire migratory properties and transit into a myofibroblast-like phenotype capable of synthesizing ECM components [55].

### 2.1. Common Mediators in Inflammation and Fibrogenesis

Both inflammation and fibrogenesis are complex processes in which numerous pro-inflammatory and pro-fibrogenic mediators can be involved. On one hand, there are chemokines considered to regulate immune cell entry into the inflamed tissue. On the other hand, there is a convolute of cytokines that modulate important functions in the inflammatory response and in the progression of inflammation to fibrosis. In addition, enhanced production of non-peptidic factors, such as reactive oxygen species (ROS), oxidized lipid mediators, and acetaldehyde, contribute to endothelial dysfunction and tissue injury during inflammation [60].

Chemokines are a group of small, mostly basic, structurally-related molecules that modulate the trafficking of leukocytes and have critical immunological functions [61]. In the last few decades, different chemokines have emerged as important molecules whose importance extends far beyond their most famous function as inflammatory mediators [61]. For several chemokines, the exact functions in different organs were identified, while others can act in an organ-independent manner [55]. Prototypically, the C-C motif chemokine 2 (CCL2), also known as monocyte chemoattractant protein-1 (MCP-1), can activate tissue macrophages and fibroblasts during the inflammatory response in many organs. Chemokines are produced by a broad range of cells and can act in an autocrine and paracrine manner. They bind to surface-exposed receptors and transmit their signals via specific intracellular signaling pathways that modulate the expression or activity of downstream targets. Besides TGF-β and PDGF that were already discussed above, different interleukins (ILs) possessing pleiotropic activities in the innate and adaptive immune response critically contribute to the onset and progression of inflammatory responses. However, their biological activity and impact in different disease settings is more variable than the effects mediated by TGF-β and PDGF. In addition, individual members of the IL family can have overlapping, but also distinct, biological activities and may exert pro- or anti-inflammatory activities [62]. Another important cytokine in the process of inflammation and fibrosis is the tumor necrosis factor-α (TNF-α), which belongs to the TNF family composed of about 20 different proteins and is produced by macrophages, amongst others [63]. The activation of its cognate receptors (TNFR1 and TNFR2) stimulates two different signaling pathways. While TNFR1 activates NF-κB and is associated with apoptosis, TNFR2 mainly triggers cell survival pathways [64]. Therefore, it is obvious that this dual activity makes TNF-α one of the key switches that determines the outcome of an inflammatory response.

Some of the biological activities of ROS are directly linked to its potential to induce TGF-β expression and activity [65]. Under physiological conditions, NADPH oxidase-derived ROS are essential modulators of signal transduction pathways that control cell growth, proliferation, migration, differentiation, apoptosis, diverse biochemical pathways, and immune responses [66]. However, elevated ROS quantities cause direct irreversible oxidative damage of all kinds of biomolecules, thereby contributing to various pathological alterations, including inflammation [66]. In particular, ROS is known to induce parenchymal cell necrosis and apoptosis and stimulate the production and release of pro-fibrogenic signaling molecules [55].

Similarly, oxidized (phospho-)lipids, such as oxidized phosphatidylcholine, can be formed and accumulate in macrophages during fibrogenesis [67]. The oxidized products promote M2 polarization of macrophages and the enhanced production of TGF-β, thereby critically contributing to fibrogenic signaling cascades. A direct effect of acetaldehyde on type I collagen expression in HSCs is mediated through acetaldehyde-responsive elements (AcRE), which are co-localized with the TGF-β-responsive element [68]. Although the mechanisms leading to an increase of collagen expression by TGF-β and acetaldehyde rely on the formation of H_2_O_2_, the kinetics of these mediators in triggering collagen expression are different. Therefore, it was assumed that early acetaldehyde-dependent events induce TGF-β expression and create an H_2_O_2_-dependent autocrine loop that amplifies the fibrogenic process [68].

More recently, it was realized that parenchymal cells under inflammatory conditions can form extracellular membranous vesicles (exosomes) that are generated by inward budding of the plasma membrane into early endosomes and multivesicular endosomes [69]. The cargo of these particles can contain diverse molecules (proteins, mRNA, microRNA, DNA, lipids), which can be transferred to distant recipient cells. Although their precise function in the transmission of signals between the different cells is not fully understood, first reports have demonstrated that in alcoholic hepatitis, hepatocyte-derived exosomes contain different microRNAs that induce a hyperinflammatory phenotype in monocytes/macrophages [70]. Similarly, a high-fat diet in rats increased the number of circulating extracellular vesicles that promote inflammation [71]. These pilot studies confirm the assumption that exosomes can mediate the communication between donor and target cells and reprogram the cells involved in inflammation. Intriguingly, recent data by Metcalfe et al. [72] showed the generation of extracellular vesicles (EVs) with an MC signature in patients with SM, which could transfer KIT to an HSC line eliciting proliferation, cytokine production, and differentiation.

### 2.2. Liver Fibrogenesis: A Number of Different Cell Types Contributing to the Initiation and Progression of Fibrosis

In the liver, viral infections (hepatitis), metabolic diseases, cholestasis, parasites, drugs, alcohol, genetic determinants, and a variety of environmental factors can lead to the initiation and progression of fibrogenesis. Like in many other organs, the pathogenic sequence begins with parenchymal cell destruction and inflammation. Hepatocytes, building 70–85% of the main parenchymal tissue, can metabolize, detoxify, and inactivate exogenous compounds. However, if the acute or chronic exposure to a toxicant is too high, the cellular phenotype of these cells becomes detrimentally altered. Cell necrosis occurs, causing many factors to leak out of injured cells, resulting in the activation of liver-resident macrophages, designated as Kupffer cells. In addition, the damaged tissue is infiltrated by various kinds of lymphocytes, which further increase the concentration of pro-inflammatory and pro-fibrotic mediators. In particular, the concentrations of TGF-β and PDGF increase. This, in turn, leads to the activation and propagation of HSCs that lose their quiescent phenotype and transdifferentiate into proliferative and extracellular matrix-producing MFBs [73]. In addition, portal fibroblasts, comprising a small population of the fibroblast cell lineage surrounding the portal vein to maintain the integrity of the portal tract, acquire a myofibroblast-like phenotype, start to proliferate, and synthesize extracellular matrix. Moreover, mesenchymal progenitor cells and fibrocytes recruited from the bone marrow are other sources of cells contributing to the generation of excessive scar formation [73]. Also, liver sinusoidal endothelial cells (LSEC) forming the wall of the hepatic sinusoids lose their fenestrae during hepatic fibrosis and form a basement membrane, preventing the physiological bidirectional exchange of molecules between hepatocytes and hepatic blood sinusoids. This corroborates with a significant synthesis and release of soluble factors, such as TGF-β and PDGF, again triggering the fibrogenic response.

However, it should be mentioned that fibrogenesis is not a unidirectional path. Regression or even full resolution can be achieved by the withdrawal of the injurious agent and is regularly seen in patients undergoing successful causative treatment of their underlying disease [74].

### 2.3. Mouse Models of Liver Fibrosis: What Can be Learned for Human Disease?

In hepatology research, preclinical mouse models are still essential for analyzing complex disease-associated changes to unravel cellular reactions, signaling pathways, and networks, as well as for the preclinical testing of novel therapeutic useful anti-inflammatory or anti-fibrotic drugs [62]. The usage of mice is majorly attributable to the fact that mice are inexpensive, can be bred in large quantities on an inbred genetic background helping to establish reproducible results, and having general features in anatomy and biology that are similar to humans [62]. Nowadays, a large number of well-established injury models are commonly used in experimental and molecular hepatology. For most of these models, we have recently published standard operating protocols (SOPs) that provide information about the scientific background, treatment, duration, and burden [75,76,77,78,79,80,81,82,83,84].

In Table 1, we exclusively list relevant experimental models that have been the focus of liver-related MC research.

#### 2.3.1. Chemical-Based Injury Models

Most common are models in which a toxic chemical substance (hepatotoxin), such as carbon tetrachloride (CCl_4_) or thioacetamide (TAA), is repeatedly administrated over a longer period [75,77]. At early time points, the applied toxins induce acute inflammation, while long-term intoxication results in robust and highly reproducible fibrosis, cirrhosis, or even HCC. During the acute phase, Kupffer cells induce an inflammatory response associated with secretion of chemokines, cytokines, and many other pro-inflammatory factors. This milieu attracts phagocytic active white blood cells (monocytes, neutrophils, and lymphocytes), stimulating parenchymal necrosis. While short-term injections of toxins are often used to study liver regeneration after toxic injury, the prolonged application of these compounds are the most widespread models for analyzing hepatic fibrogenesis. Toxic drugs much less frequently used in liver fibrosis research are dialkyl nitrosamines, such as dimethylnitrosamine (DMN) and diethylnitrosamine (DEN). These are hepatocarcinogens that provoke severe liver damage in mice when given parenterally or orally [81]. In the acute phase, these compounds provoke intense neutrophilic infiltration and extensive centrilobular haemorrhagic necrosis. When applied for longer periods, both DMN and DEN induce massive bile duct proliferation, ending in fibrosis, bridging necrosis, and in liver cancer [81]. Based on their mutagenic and carcinogenic properties, DMN and DEN are frequently used in translational research by investigators interested in recapitulating the multi-stage process of human liver carcinogenesis or the formation of HCC.

The application of LPS alone or in combination with other substances such as D-galactosamine (D-GalN), *Mycobacterium bovis* bacille Calmette–Guérin (BCG), or the diptheroid *Corynebacterium parvum* is frequently used to induce severe hepatic inflammation or lethal hepatitis [79]. LPS is composed of lipid- and polysaccharide-containing moieties found in the outer membrane of Gram-negative bacteria. It acts as a PAMP that is recognized by TLR4 after binding to a special LPS-binding protein in the serum. Among others, the NF-κB pathway is activated and provokes the strong activation of Kupffer cells [79]. The simultaneous application of substances, such as the amino sugar D-GalN, that inhibits synthesis of different macromolecules and induces hepatocyte damage and formation of intracellular ROS leads to a several-thousand-fold increased susceptibility toward LPS. Single injection of this endotoxin is the most commonly used toxemia model and is suitable for inducing a shock-like state. However, compared with mice, humans are more sensitive to LPS. In humans, small traces of gut-derived LPS resulting from leakages in the gut epithelium entering the portal circulation are already adequate to establish severe hepatic inflammation.

#### 2.3.2. Surgery-Based Injury Models

In mice, ligation of the common bile duct causes obstructive cholestatic injury and periportal fibrosis [82,83]. This procedure, known as bile duct ligation (BDL), provokes time-dependent morphological and structural changes, which is combined with elevated serum activities of aspartate aminotransferase (AST), alanine aminotransferase (ALT), γ-glutamyltransferase (γ-GT), alkaline phosphatase (AP), and lactate dehydrogenase (LDH) [82]. Over time, ongoing liver damage is reflected in jaundice. Besides the typical markers that reflect inflammation and fibrogenesis, the large subset of cytokeratins that can be detected by immunohistochemistry reflects the occurrence of the ductular reaction. This model is often taken to recapitulate diverse forms of human congenital and acquired cholestasis.

#### 2.3.3. Genetic Models

A plenitude of genetically-modified models in which genes are overexpressed or silenced, or in which special transgenes allow sophisticated cell fate tracing experiments, are frequently used [86,87]. Prototypically, mice carrying a homozygous disruption of the *Mdr2* gene encoding the multidrug resistance 2 protein (a drug-transporting P-glycoprotein) develop a liver disease that appears to be caused by the complete inability of the liver to secrete phospholipids into the bile [85]. This results in an accumulation of toxic bile acids in the biliary canaliculus, which damages hepatocytes and cholangiocytes, subsequently inducing liver inflammation. This model is therefore ideally suited to analyze human diseases that are associated with alterations in bile acid flow or synthesis, such as primary sclerosing cholangitis (PSC) or primary biliary cholangitis (PBC).

#### 2.3.4. Mouse Models in Translational Research of Human Liver Diseases

**Figure 1:**
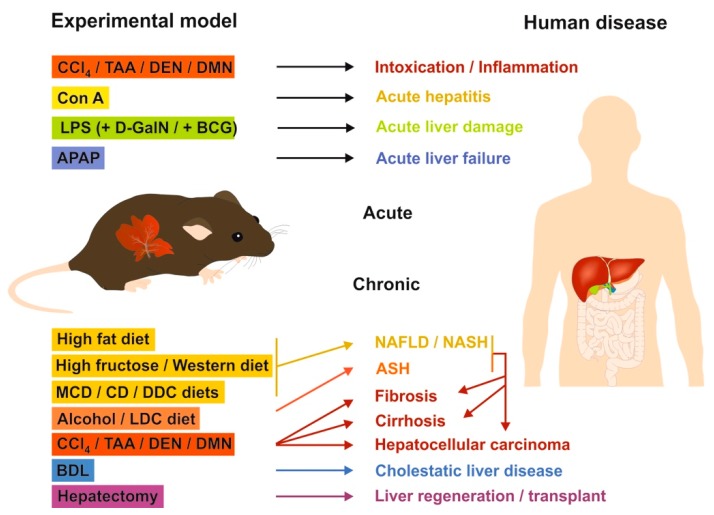
Mouse models mimicking human liver disease. The application of different hepatotoxins, feeding of special diets, and the execution of surgical procedures in mice are extensively used to explore basic pathophysiological mechanisms of human hepatic disease. In addition to these treatments, transgenic, knockout, and knock-in mice serve as tools to gain insight into human liver disease. Abbreviations used are: APAP, *N*-acetyl-p-aminophenol (acetaminophen); ASH, alcoholic steatohepatitis; BCG, bacillus Calmette–Guérin; BDL, bile duct ligation; CCl_4_, carbon tetrachloride; CD, choline-deficient; Con A, Concanavalin A; DDC, 3,5-diethoxycarbonyl-1,4-dihydrocollidine; D-GalN, D-galactosamine; DEN, diethylnitrosamine; DMN, dimethylnitrosamine; LDC, Lieber-DeCarli; LPS, lipopolysaccharide; MCD, methionine- and choline-deficient; NAFLD, non-alcoholic fatty liver disease; NASH, non-alcoholic steatohepatitis; TAA, thioacetamide.

The above-mentioned animal models have become increasingly popular in recent years. In particular, each model is taken by investigators to analyze different aspects of human liver disease ranging from intoxication, inflammation, liver damage, liver failure, NAFLD/NASH, ASH, fibrosis, cirrhosis, hepatocellular carcinoma, cholestatic liver disease, to liver regeneration (Figure 1).

A study comparing mouse models of inflammation with the corresponding human disease demonstrated that studying disease in patients is generally much more complex than studying experimental model systems, suggesting that the extrapolation of preclinical data to the human situation must be critically questioned [88]. However, despite this partly justified criticism, animal models in hepatology research are still invaluable tools in understanding molecular pathological aspects in the initiation and progression of liver disease.

## 3. Functional Interaction of Mast Cells and Other Cell Types Relevant for Fibrosis

MCs have been realized as modulators of fibrotic processes in different organ systems. In line with a potential role of MCs in liver fibrosis is the finding that portal fibrosis is a frequent complication in SM. In this setting, MC numbers are highly increased in the liver [89,90]. It is a common feature that MC numbers increase in tissues during the process of fibrogenesis and the expansion of the MC pool in the liver is a common characteristic of liver fibrosis in humans and animal models due to damage by chemical toxins, viral infections, and cholestasis [91,92,93,94]. In fact, the physiological number of MCs in the liver is sparse and MC numbers strongly increase upon different pathological conditions [4,95,96,97]. According to Gentek et al., such an increase in MC numbers might occur via proliferation/differentiation of long-lived tissue-resident precursors [15].

The localization of MCs in the liver is mostly restricted to portal tracts and MCs are recruited to these areas under pathological conditions [98]. They are most likely absent from sinusoids and liver parenchyma [95,96]. However, in human liver, ≈10% of MCs have a perisinusoidal location [92]. Because of the prominent appearance in the portal tracts, most studies have been performed in the setting of cholangiopathies [99]. In those models, the inflammatory and fibrotic responses emanate from the portal fields based on a biliary reaction [82,83]. Due to this, the focus has been put on the interaction of MCs with cholangiocytes and to the pathways driving fibrogenic responses in HSCs/portal (myo)fibroblasts (see below). Nevertheless, there are a few reports addressing the crosstalk of MCs with other immune cells, i.e., Kupffer cells (see below) and the main cell type in the liver, i.e., the hepatocytes. For the latter, it was shown in vitro that syngenic BMMCs co-cultured with primary hepatocytes increases hepatocyte proliferation [100].

The infiltration (of myeloid lineage-derived cells from the bone marrow) and accumulation (differentiation of liver resident precursor cells, which can be differentiated in the presence of SCF) of MCs in the insulted liver has been shown in several studies and is unquestionable [101]. Even more, the isolation of MCs from liver of BDL-treated animals has been established [102]. It should be noted that not only the presence of MCs itself is of vital importance, but also the type of MCs within the liver (i.e., CTMCs or MMCs), when the functional interplay of cells is considered. General responses regulated by FcεRI or KIT are common to all MCs, whereas the contribution of effector enzymes for crosstalk with other cells varies in between different MC types [103]. A study in human liver tissue demonstrated that the amount of tryptase/chymase-positive MCs is higher than tryptase-positive cells in healthy and chronically diseased livers [104]. The cells isolated from BDL rats were chymase- and tryptase-positive, reflecting that these cells most likely represent CTMCs [102].

### 3.1. SCF–KIT Axis as Chemotactic Guidance

Initial work aimed to identify the factor(s) governing MC infiltration (chemotaxis). There have been several factors identified that govern MC migration [105]. Those include two key factors that act as a chemoattractant for MCs and that are important in the process of liver fibrosis. The first one and the most potent is TGF-β1, which is increasingly expressed in the liver under fibrogenic conditions [106,107]. However, aside migration/chemotaxis, TGF-β1 has an impact on several critical functions of MC biology, including proliferation, apoptosis, effector synthesis, and degranulation (see below). In the second line, stem cell factor (SCF), which is an essential factor for MC proliferation, survival, and differentiation is also a potent chemoattractant for MCs and circulating MC progenitors [108,109,110]. The SCF transcript can be differentially spliced, resulting in alternative transcripts, which differ in the presence or absence of exon 6. Both encoded proteins are membrane-bound but the longer one, including exon 6, can be more easily proteolytically cleaved to generate soluble SCF [111]. SCF is produced by fibroblasts and endothelial cells, and both the membrane-bound and soluble SCF bind to the surface tyrosine kinase membrane receptor KIT (CD117), which leads to the recruitment of MC progenitors and activation of MCs in the tissue.

Recruitment of MCs to the liver requires the liberation of progenitors into the circulation and entry into the tissue at the destination site. In this scenario, extravasation of circulating MC progenitors is facilitated by the interaction of MC progenitors with endothelial cells [11]. This contact can be mediated by the α4β7 integrin, as has been shown for murine MC homing/recruitment to the small intestine [11]. For human MCs, attachment to endothelial cells was shown to rely on the α4β1 integrin subunits [112]. As mentioned above, endothelial cells express the MC chemoattractant SCF.

In order to substantiate SCF as the basis for MC recruitment during liver disease, the expression of SCF in normal as well as diseased livers from patients suffering from PBC or PSC was analyzed using RT-qPCR and ELISA showing that SCF was increased. The corresponding SCF mRNA could be detected in human and rat primary HSCs. The expression of SCF increased during the culture, activation, and transdifferentiation of primary rat HSCs. In turn, isolated human skin MCs adhered in co-culture to HSC monolayer cells, most likely via membrane-bound SCF, since this interaction could be blocked by anti-SCF antibodies [110]. HSCs do not constitutively express SCF, but it can be induced by MCs in a TNF-α-dependent fashion [113]. In addition to HSCs, SCF is produced by keratinocytes, airway epithelial cells, and endothelial cells. In a recent paper, another critical player in fibrosis, i.e., cholangiocytes, have been shown to express and secrete SCF, in contrast to hepatocytes, which do not express SCF [114]. Inhibition of SCF expression in *Mdr2*^−/−^ mice decreases MC recruitment in vivo. In vitro deprivation of SCF in cholangiocytes reduces MC migration and HSC activation. As a consequence, histamine levels, biliary reaction, and fibrosis were reduced in *Mdr2*^−/−^ mice [115]. Moreover, it has been shown that the SCF-mediated migration of MCs depends on the activity of the sheddase/metallproteinase ADAM10 [115].

In addition to migration/recruitment, recombinant human SCF, as well as NIH3T3 fibroblasts (in co-culture), have the capability to induce the differentiation of MC precursors isolated from human fetal liver [116,117,118]. Indeed, mouse NIH3T3 fibroblast cells express SCF constitutively [119]. Analysis of glucocorticoids (dexamethasone) on a co-culture of MCs with NIH3T3 fibroblasts or HSCs showed that dexamethasone blocks NIH3T3-mediated MC proliferation, while it has no effect on HSC-mediated MC proliferation, because the latter induces SCF expression in HSC at a post-translational level [119].

In a recent work, Kim and colleagues analyzed extracellular vesicles (EVs) of patients suffering from SM. They found that EVs show an MC signature, including the proteins tryptase, FcεRI, and KIT [72]. Incubation of HSCs in vitro with these purified EVs led to the transfer of functional KIT to stellate cells. This transfer caused an increase in HSC proliferation, cytokine production, and differentiation. These effects were blocked by KIT inhibition, while in in vivo experiments, the application of EVs to mice increased α-smooth muscle actin (α-SMA) production and the presence of human KIT in mouse HSCs. These results suggest that KIT expression/presence on HSCs causes activation of these cells [72]. A mutual involvement of Kupffer cells in the recruitment of MCs to the liver was demonstrated in an endotoxin liver injury model that causes hepatocyte damage, an inflammatory reaction, and MC infiltration. Treatment of those animals with an agonist for the liver X receptor (LXR; GW3965), highly expressed in Kupffer cells, leads to reduced liver affection and reduction in MC count [120]. If this effect is mediated by Kupffer cells, or if the agonist has a direct impact on MCs is questionable since the LXR agonist GW3965 directly blocks at least inflammatory cytokine production of BMMC [121].

### 3.2. Impact of Mast Cells and Mediators on Liver Disease and Individual Cells

**Figure 2:**
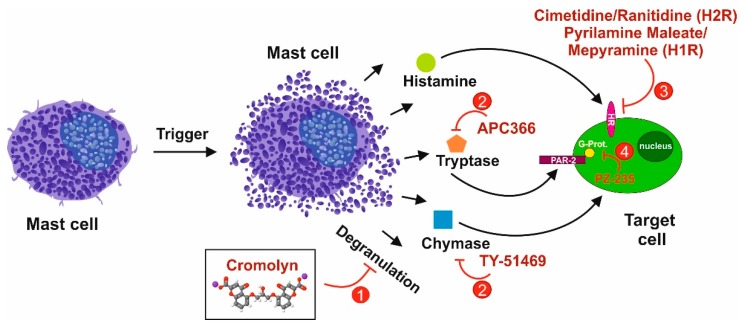
Regulation of mast cell (MC) effector functions. Upon liver damage, MCs increase in the liver tissue and degranulation is triggered by environmental factors. The released MC effectors modulate target cell functions in receptive cells, such as hepatic stellate cells, portal myofibroblasts, and cholangiocytes. There are several possible ways to interfere with MC effector functions: (1) MC stabilizers like cromolyn block the general release of MC granula, and inhibitors can inactivate liberated (2) proteases, (3) block different types of histamine receptors, or (4) can modulate downstream signaling in target cells. Abbreviations used are: APC366, *N*-(1-hydroxy-2-naphthoyl)-L-arginyl-L-prolinamide; G-Prot., G protein; HR, histamine receptor; H1R, histamine receptor H1; H2R, histamine receptor H2; PAR-2, protease-activator receptor-2; PZ-235, P2pal-18S cell-penetrating pepducin targeting the intracellular i3 loop of PAR-2; TY-51469, 2-[4-[(5-fluoro-3-methyl-1-benzothiophen-2-yl)sulfonylamino]-3-methylsulfonylphenyl]-1,3-thiazole-4-carboxylic acid.

Once MCs have infiltrated into the interstitial tissue of the portal tracts, MCs most likely get activated by environmental factors. Through the process of hyperplasia and degranulation (Figure 2), effector molecules are liberated, which in turn act on surrounding cells, including bile duct epithelial cells, HSCs, portal fibroblasts, and resident Kupffer cells. In addition, these mediators are further triggers that recruit circulating immune cells to the site of injury. On the other hand, it has been described that the contents liberated after degranulation of MCs can act in an endocrine fashion via the bile ductules/bile showing long-range effects [122]. In addition to the “classical” degranulation, a cell-to-cell interaction has been described and termed transgranulation. This interaction occurs with MCs and fibroblasts/vascular endothelial cells and describes the direct transfer of granules from MCs via pseudopodia to the communicating cell. This process has been observed in vitro as well as in vivo [123].

In kinetic experiments using CCl_4_ intoxication, MC infiltration was associated with individual stages of fibrosis. At the time points with significant MC infiltration, HSCs were not yet activated. Therefore, the authors concluded that MCs are indicators of acute inflammation [124]. Work contributed by Takeshita [125] implied that after BDL surgery in rats, MC amounts increase in the liver but not that much in the portal fields. In contrast, there is a strong but transient increase in MC number after recanalization of the bile duct [125]. The authors concluded that MCs are not involved in the early phase of fibrosis, but rather in the apoptosis of biliary epithelial cells and removal of bile ductules after recanalization [125]. However, further data from the time-resolved analysis of BDL or DMN-treated rats imply that MCs do play a role in the fibrotic phase of liver disease [95,126]. Results from CCl_4_-treated rats revealed that myofibroblasts and macrophages increase in number during the first ten weeks, whereas MCs increase constantly over the period peeking in number at week 14 [127]. Nevertheless, although the function of MCs in the process of fibrosis is not completely resolved, it is common sense that most of the studies performed so far showed that MCs fulfill profibrogenic functions in diseased liver (see below).

Only one of the early works performed by Sugihara et al. implied that MCs have no consequence in the development of liver fibrosis in wild-type or MC-deprived *Kit^Ws/Ws^* rats and in wild-type or MC-deprived *Kit^W/Wv^* mice [128]. The animals were subjected to BDL and treated with CCl_4_ or porcine serum (rats) and CCl_4_ or BDL (mice) to induce portal tract (BDL, serum) or parenchymal fibrosis (CCl_4_). The treatments lasted 21 days and were evaluated using a hydroxyproline measurement for collagen deposition and Alcian blue stain to detect MCs. Since the early time points (less than 21 days) were not taken into account, the reaction of the biliary tree and infiltration of MCs during the early events of fibrosis were not evaluated. All treatments caused fibrosis and increased quantities of MCs. However, although the overall MC quantities were approximately 20-fold higher in wild-type than in *Kit^Ws/Ws^* animals, the degree of fibrosis was equal in both groups. Therefore, the authors concluded that MCs have no role in fibrosis [128]. On the other hand, a recent paper analyzing the influence of MCs on fibrosis in a direct co-culture system or an indirect co-culture system composed of a human MC line and primary MCs with the human HSC line LX2 showed that soluble factor(s) derived from MCs caused a decrease in ECM/collagen I abundance in the presence or absence of TGF-β, IFN-α, or IL-10 [129]. They could show by using the APC366 tryptase inhibitor and the broad range inhibitor Chymostatin, that the key MC proteases tryptase and chymase are most likely involved in collagen I degradation. In addition, the human leukocyte antigen G (HLA-G), representing a histocompatibility antigen, is increasingly expressed in MCs when co-cultured with HSCs. This is accompanied by a significant decrease in collagen production, irrespectively of whether cells were stimulated with TGF-β, IL-10, or IFN-α, or left untreated [129]. In their study, the authors proposed MC proteases as critical mediators of collagen degradation. HLA-G has been studied by the same authors before in the setting of chronic HCV infection. They could show that HLA-G in situ is produced by MCs and is up-regulated upon stimulation with IL-10 and IFN-α, leading to the recruitment of T-lymphocytes and NK cells [130].

Nevertheless, taking into account data of MC involvement in fibrosis in lung, kidney and heart, there is a great body of evidence that MCs play a pro-fibrogenic role in liver fibrosis [131,132,133,134,135].

### 3.3. BDL-Induced Cholestasis in Mast Cell-Depleted Kit^W-sh/W-sh^ Mice

In order to delineate pro-fibrogenic effects of MCs in more detail, the crosstalk of MCs with other liver resident and infiltrating cells has been analyzed in murine models of cholestatic liver injury. Due to the localization of MCs in the portal tracts of the liver, different animal models and human biopsies have been analyzed (BDL, *Mdr2*^−/−^ mice, porcine serum injection, PBC and PSC samples) [97,136]. The animal models develop portal fibrosis and a bile duct reaction encompassing bile duct proliferation (cholangiocyte proliferation), bile duct hyperplasia, and angiogenesis as critical features [137].

In order to delineate the effects of MC absence in vivo on processes induced by cholestasis, wild-type and MC-depleted mice (*Kit^W-sh/W-sh^*) underwent BDL surgery. Wild-type mice subjected to BDL displayed increased focal necrosis (infarct) and portal inflammatory changes that are consistent with obstruction. In BDL *Kit^W-sh/W-sh^* mice, the degree of focal necrosis (infarct) was reduced. Liver enzymes were significantly upregulated in wild-type mice after BDL, but this was completely ablated in BDL *Kit^W-sh/W-sh^* mice, suggesting diminished hepatocyte damage in the absence of MCs. Biliary and cholangiocyte proliferation was reduced in BDL *Kit^W-sh/W-sh^* mice. With respect to HSC function, the collagen deposition was dramatically reduced in BDL *Kit^W-sh/W-sh^* mice, as monitored by Fast Green/Sirius Red staining. In agreement, the expression of α-SMA, fibronectin-1, collagen I type 1α, and SYP-9 as activation markers of HSCs were decreased in BDL *Kit^W-sh/W-sh^* mice. In addition, there was a significant reduction in both TGF-β1 expression and secretion in BDL *Kit^W-sh/W-sh^* mice, while TGF-β1 levels were increased in *Kit^W-sh/W-sh^* mice injected with cultured MCs, supporting the concept that MCs are an important source of TGF-β1 during fibrosis progression [138]. TGF-β1 expression and secretion by antigen-stimulated MCs has been shown to induce proliferation, collagen Iα1, and monocyte chemoattractant protein-1 (MCP-1) expression in fibroblasts [139,140,141].

As a confirmation of the aforementioned findings, HSCs were cultured in vitro with conditioned supernatants derived from cholangiocytes isolated from BDL treated *Kit^W-sh/W-sh^* animals. Only when the cholangiocytes were isolated from *Kit^W-sh/W-sh^* animals supplemented with MCs, the corresponding supernatants have the potential to activate HSCs, as displayed by higher α-SMA expression. Those results imply that the presence of MCs triggers cholangiocytes to secrete factors that are able to cause HSC activation in vitro [138].

### 3.4. Tryptase/Protease-Activated Receptor-2 Axis

One of the prominent effectors secreted by MCs upon degranulation is the protease tryptase. In contrast to chymase, tryptase is found in both human MCs (MC_TC_ and MC_T_) [142]. On the other hand, in the mouse, tryptase is only expressed in CTMCs but not in MMCs [143]. Tryptase, in addition to trypsin and coagulation factors, activates the protease-activated receptor (PAR)-2 via limited proteolysis of its N-terminus. Protease-activator receptor-2 (PAR-2) is expressed in hepatic endothelial cells, Kupffer cells, hepatocytes, bile duct epithelial cells, and HSCs. The expression of PAR-2 in HSCs is increased during liver fibrosis [144]. In agreement, the analysis of PAR-1 and PAR-2 expression in isolated rat HSCs showed that mRNA expression increases during the transdifferentiation of HSCs to MFBs in culture [145].

In vivo, a PAR-2 knockout had no impact on initial liver fibrosis after 5 weeks of CCl_4_ treatment but prevented the progression of fibrosis with sustained CCl_4_ treatment after 8 weeks, as reflected by lower hydroxyproline content. A similar behavior was seen in the expression of α-SMA, TGF-β1, matrix metalloproteinase (MMP)-2 (gelatinase A), and tissue inhibitor of metalloproteinase (TIMP)-1. The expression of PAR-1 compensates for PAR-2 deficiency, but only in the early phase (5 weeks) and not in the late phase (8 weeks). Since activation of PAR-2 leads to the recruitment and activation of macrophages, their presence at the different time points were quantified. F4/80^+^ macrophages are reduced after 5 and 8 weeks of treatment, whereas CD68^+^ macrophages were only reduced at 8 weeks. These observations are consistent with a role for PAR-2 in the recruitment and later activation of macrophages in CCl_4_-induced hepatic fibrosis. Therefore, these in vivo data implicate that PAR-2 is involved in the activation of HSCs, TGF-β1 synthesis, and ECM deposition, as well as macrophage recruitment.

To substantiate these findings with in vitro data, human HSC LX2 cells were induced for 48 h with the peptidic PAR-2 agonist SLIGKV. This stimulation induced proliferation of HSCs similar to that of PDGF, representing the most potent inducer of HSC proliferation. The PAR-2 agonist also led to an increase in collagen I and TGF-β1 production. Those effects were also observed when using a PAR-1 agonist, but the induction achieved with the PAR-1 and PAR-2 agonists on collagen I and TGF-β1 protein content were not additive [144]. Agonists for PAR-1, such as thrombin and SFFLRN, or agonists for PAR-2, such as tryptase and SLIGRL, induced a proliferative response in rat HSCs. Due to the sensitivity of this response to PD98059, it is most likely that the observed effects are mediated by ERK1/2 activation. In addition, tryptase and SLIGRL increased the collagen I secretion by HSCs [145]. In a more recent study, MC tryptase induced the activity of PAR-2 and increased HSC activation and proliferation, thus promoting hepatic fibrosis and implying that MCs interact directly with HSCs to drive fibrosis. The application of the MC tryptase inhibitor APC366 in BDL-induced hepatic fibrosis in rats showed that APC366 reduced hepatic fibrosis scores, collagen content, and serum biochemical parameters. Moreover, the reduced fibrosis was associated with a decreased expression of PAR-2 and α-SMA. Therefore, it was suggested that MC tryptase induces PAR-2 activation to augment HSC proliferation and promote hepatic fibrosis in rats [146].

### 3.5. Chymase as an Interface for the Activation of Several Pro-fibrogenic Pathways

The MC chymase activity is increased in the livers of patients with fibrosis or cirrhosis and there is a significant correlation between the chymase level and the degree of fibrosis [147]. Chymase can cleave the Phe8–His9 bond of the non-bioactive peptide angiotensin I (Ang I), forming its bioactive peptide angiotensin II (Ang II) in mammalian tissues including humans [148]. Ang II also induced hepatic fibrosis via the induction of α-SMA in HSCs [149]. Ang II and its angiotensin receptor 1 (AT1) are involved in the fibrotic process. In human fibrosis, MC chymase expression is increased, which is coupled with an increased expression of myofibroblast Ang II receptor, AT1. The receptor is expressed on vascular smooth muscle cells, HSCs, portal myofibroblasts, and hepatocytes. During cirrhosis, the expression of AT1 is increased in fibrotic septa and vessels. Ang II and chymase can bind to AT1 on HSCs and MFBs in fibrotic septa and promote fibrosis [150]. The effect of chymase on isolated HSCs reveals that the protease enhances HSC proliferation, TGF-β1/α-SMA protein expression, and collagen I formation [151]. In addition to the activation of Ang II, chymase was shown to enzymatically cleave the precursors of MMP-9 (pro-gelatinase B), TGF-β, and collagen I, leading to their biologically active forms [152,153,154]. Furthermore, the enzymatic function of MC-released chymase can produce soluble SCF via enzymatic cleavage of the membrane-bound form of SCF on stromal cells, which induces the formation of mature MCs from immature MCs via the stimulation of KIT [155]. Due to the multiple roles of chymase, which may promote organ fibrosis, this enzyme is a promising target for anti-fibrotic agents [156].

### 3.6. The Histamine/Histamin Receptor Axis

In the setting of cholangiocarcinoma (CCA), MCs within the tumor environment release histamine, which increases CCA progression and angiogenesis. Conversely, cholangiocytes secrete SCF, which binds and activates the MC growth factor receptor KIT. Cholangiocytes express histidine decarboxylase and its inhibition reduces CCA growth [157]. In the study by Johnson et al., MCs were detected in human CCA biopsies and their recruitment is mediated via SCF in the tumor microenvironment stimulating CCA growth. In xenograft tumor mice treated with the MC stabilizer cromolyn sodium, MC infiltration and tumor growth decreased. Inhibition of SCF in CCA blocked MC migration and MC/EMT/ECM in CCA [157]. MCs migrate into the CCA tumor microenvironment via KIT/SCF and increase tumor progression, angiogenesis, and ECM degradation [157]. In a further study analyzing histamine function, *Mdr2*^−/−^ mice (PSC model) were treated with cromolyn. This treatment reduced MC protease mMCPT-1, as well as serum histamine. In agreement, human PSC samples also showed a robust expression of MC markers. Necrosis, lobular damage, and bile duct reactions, such as intrahepatic biliary mass and cholangiocyte proliferation, were also reduced via MC silencing using cromolyn in *Mdr2*^−/−^ mice.

As a confirmation of the in vivo analysis, co-culture experiments of cholangiocytes with MCs induced cholangiocyte proliferation, α-SMA, fibronectin-1, and TGF-β1 production, functions that are blocked in MC depleted using an siRNA approach targeting histidine decarboxylase. In a similar experiment, MCs induced PCNA, α-SMA, and fibronectin-1 in human HSC, and these effects were also blocked by the knockdown of HDC in MCs. These in vitro results imply that cholangiocyte and HSC responses to MCs are dependent on the histamine secreted by MCs [158]. Nevertheless, the in vivo results using cromolyn have to be handled with caution because this substance directly affects HSCs and hepatocytes. Cromolyn was originally developed as an MC stabilizer. However, the activation and collagen accumulation for the HSC cell lines LX2 and HSC-T6 were reduced by 50% after cromolyn treatment at a low concentration without signs of apoptosis. Furthermore, cromolyn treatment compromised the TGF-β-induced epithelial to mesenchymal transition and replicative senescence rate of hepatocytes, which are generally associated with fibrogenesis. Taken together, cromolyn may be the basis for an effective cure for fibrosis and cirrhosis because it targets both HSCs and hepatocytes [159].

In a more reliable/specific setting to analyze the function of MC histamine, Kennedy et al. used histamine receptor antagonists. These reduced the lobular damage, necrosis, and inflammation in *Mdr2*^−/−^ mice. The histamine receptors H1 (H1R) and H2 (H2HR) are upregulated in cholangiocytes of *Mdr2*^−/−^ mice, as well as in human PSC and CCA. Blocking of the histamine receptor leads to decreased activation of MCs as evaluated via the detection of MC markers in *Mdr2*^−/−^ mice and in the CCA model [160]. Histamine serum levels decrease in *Mdr2*^−/−^ and CCA mice in the presence of respective blockers. Bile duct reaction, i.e., biliary proliferation and intrahepatic bile duct mass, were reduced. Treated *Mdr2*^−/−^ mice had a lower collagen deposition and lower expression of the HSC activation marker synaptophysin-9 (Syp-9). Histamine receptor blockers do not affect activation of HSCs directly [160]. The tumor growth is blocked in the CCA model and Ki67-positive proliferating cholangiocytes were reduced. In a co-culture/conditioned medium model, MCs increased the proliferation of cholangiocytes and progression of CCA, and this effect was reduced in the presence of inhibitors. In summary, histamine receptor blockers reduce PSC and CCA progression [160]. As a general feature, MCs stimulate fibroblast proliferation [161,162] and collagen synthesis [163,164] via histamine secretion. Moreover, it has been shown that MCs themselves are competent at expressing basement membrane components, including collagen IV and laminin [165].

An early study by Akiyoshi et al. reported that MCs, portal myofibroblasts, and cholinergic nerve terminals work synergistically to promote liver fibrosis [137], demonstrating that the paracrine influence from MCs is not limited to altered HSCs and cholangiocyte function.

### 3.7. TGF-β1

It has been stressed before that one of the main drivers of fibrosis is TGF-β1, which is strongly upregulated in the process of fibrosis and leads to activation and ECM production by HSCs and portal fibroblasts [166]. In addition to liver resident cells, e.g., HSCs and Kupffer cells, MCs also express and secrete TGF-β1 to promote fibrosis (see above). The pro-fibrogenic master cytokine, TGF-β1, regulates the expression of proteases and their release [167]. In contrast, MC-specific receptors controlling cellular degranulation, including KIT (ligand SCF) and FcεRI (ligand IgE), are down-regulated in vitro and in vivo by TGF-β1 in a SMAD-dependent fashion [168]. In addition, TGF-β1 suppresses IL-33-induced cytokine production and MC activation by interfering with MAP kinase phosphorylation [169]. Aside from the expression and liberation of effectors, a broad spectrum of MC functions is modulated by TGF-β1, including proliferation, cell cycle control, and apoptosis [170]. Another vital function of MC biology modulated by TGF-β is migration/chemotaxis. Interestingly, chemotaxis is not mediated by the classical SMAD pathway, but by MEK1/2 signaling [171]. Moreover, the SRC family kinase FYN plays a critical role in TGF-β1-mediated MC migration in vitro and in vivo [172]. Those observations show that in the environment of fibrosis MCs may contribute to the progression of the disease by releasing TGF-β1, affecting HSCs and portal fibroblasts. In turn, MCs also express TGF-β receptors and are targets for this ligand [107]. Nevertheless, the effects are somewhat counterbalanced, leading on one hand to chemotaxis and increased effector synthesis [107,173], and on the other hand apoptosis, blocking late stage maturation and sensor suppression [173,174,175]. Therefore, the TGF-β1 response in MCs is a double-edged sword, which largely depends on the molecular context.

**Figure 3:**
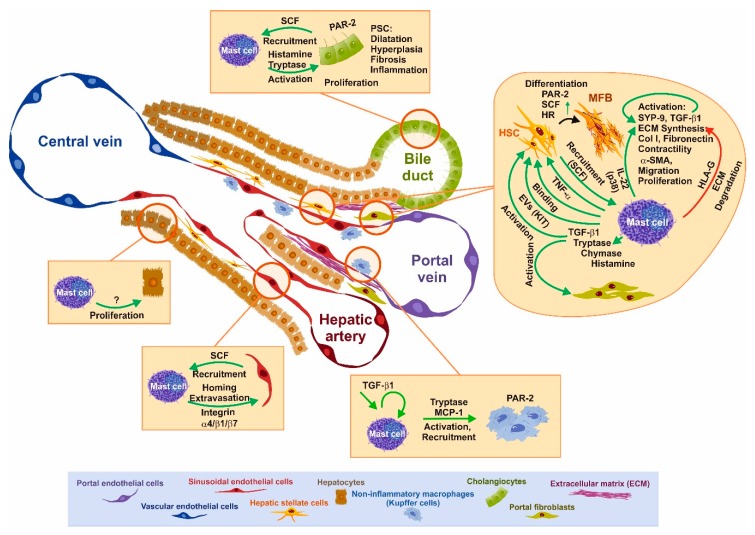
Schematic overview of known functional interactions between mast cells and liver cells in hepatic fibrogenesis. Mast cells (MCs) activation and degranulation leads to a production/release of many biological active compounds including TGF-β1, tryptase, chymase, histamine, TNF-α, and human leukocyte antigen G (HLA-G). These soluble mediators trigger the recruitment/activation of inflammatory blood cells and synthesis of an extracellular matrix (ECM) by stimulating the propagation of pro-fibrogenic cells (hepatic stellate cells, portal myofibroblasts) and inhibiting its ECM degradation. Furthermore, the compounds lead to the activation of liver-resident Kupffer cells and cholangiocyte proliferation. It is further discussed that MCs modulate parenchymal cell proliferation and biological features of endothelial cells. In addition, the cargos present in extracellular vesicles (EV) that are released from MCs into the extracellular milieu facilitate pro-fibrogenic signaling pathways in recipient cells. Abbreviations used are: α-SMA, α-smooth muscle actin; Col I, collagen type I; HLA-G, human leukocyte antigen G; HR, histamine receptor; HSC, hepatic stellate cell; IL-22, interleukin 22; MCP-1, monocyte chemoattractant protein-1; MFB, myofibroblast; PAR-2, Protease-activator receptor-2; PSC, primary sclerosing cholangitis; SCF, stem cell factor; SYP-9, synaptophysin-9; TGF-β1, transforming growth factor-β1; TNF-α, tumor necrosis factor-α.

In summary, there are many mediators released by activated MCs that contribute to liver disease by directly interfering with the recruitment and activation of inflammatory blood cells, stimulating proliferation of pro-fibrogenic cells, promoting ECM synthesis, or inhibiting its degradation (Figure 3).

## 4. Mast Cells in Human Liver Disease

Today, there is first evidence demonstrating that MCs are important cellular regulators in human liver disease. In a normal human liver, MCs are associated with the connective tissue and are mainly found along the portal tracts [98]. The number of MCs in human liver is significantly increased during the pathogenesis of PBC, PSC, bile duct obstruction, hepatitis, alcohol-induced liver injury, steatosis, steatohepatitis, congenital and non-congenital liver fibrosis, liver cancer, liver rejection upon liver transplant, and liver aging [98,176,177]. Although the cellular and sub-cellular linkages of MCs to all these diseases remain unresolved, it is most likely that these cells are critically involved in the liver’s fibrotic response to chronic inflammation. On the other side, MCs can exert immunomodulatory effects on other immune cells, thereby enhancing or suppressing the initiation, magnitude, and/or duration of immune cells within the liver, preventing diminished hepatobiliary functions during disease progression, or by acting as a first effector cell in an innate response to encounter antigens [176,177]. However, the knowledge of MC function in the human liver is still very limited and further fundamental studies are urgently needed to understand the full biological repertoire and activities mediated by these important immune cells in human liver homeostasis and disease.

## 5. Therapeutic Options

### 5.1. Selective Targeting of Mast Cells in Liver Fibrosis

“Personalized medicine” and “precision medicine” are keywords when it comes to discussions about successful patient-tailored treatment of diseases; such an endeavor first requires thorough and sophisticated diagnostic tools. On a different level, this also concerns pharmacological regulation of cellular activation states depending on the (patho-)physiological tissue niches, as well as patient comorbidities. The more sensitive cells are to environmental conditions, the more complicated the choice of beneficial pharmacological substances might be. This applies in particular to MCs, whose task is to recognize changes in micro-environmental conditions and occurrence of biological mediators and/or chemical substances. As already indicated in Section 1.2., MCs are endued with enormous heterogeneity, as well as fascinating plasticity; thus, depending on their localization and prevailing conditions, MCs as a whole express hundreds of mediators and surface receptors, complicating the choice of pharmacological treatment in a given pathophysiological setting. Hence, returning to the keyword of personalized medicine, deciphering the complexity of MC phenotypes in a given (patho-)physiological situation might enhance the ability to use MCs as “drug targets.”

**Figure 4:**
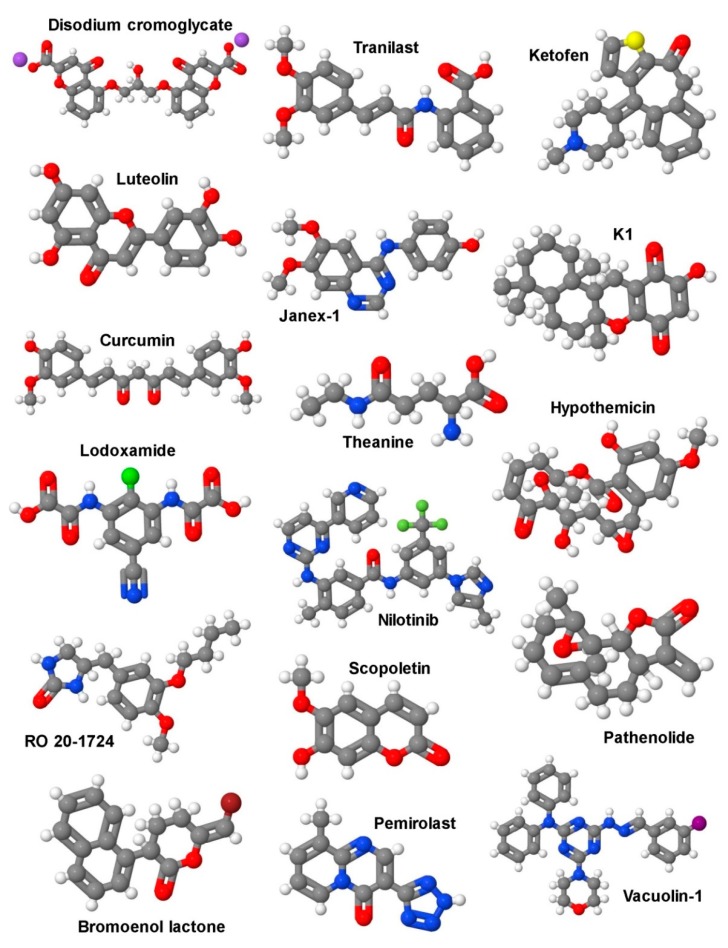
Naturally occurring and synthetic mast cell stabilizers with highly variant structures. Interestingly, some of these drugs, such as Tranilast, Luteolin, Curcumin, Theanine, Nilotinib, and others showed highly beneficial effects in experimental hepatic fibrosis [62]. Most of their activities are attributed to their antioxidant activity, capacity to prevent hepatic infiltration with circulating blood cells, potential to target pro-inflammatory and/or pro-fibrotic signaling pathways, or to influence extracellular matrix generation or turnover. However, none of these substances have been tested systematically regarding the modulation of mast cell stability in models of hepatic fibrogenesis. All structure images were prepared with Jmol (http://jmol.sourceforge.net/) using the following PubChem Compound Identification (CID) numbers: Bromoenol lactone (5940264), Curcumin (969516), disodium cromoglycate (27503), Hypothemicin (9929643), Janex-1 (3794), K1 (25211416), Ketofen (3827), Lodoxamide (44564), Luteolin (5280445), Nilotinib (644241), Pathenolide (7251185), Pemirolast (57697), RO 20-1724 (5087), Scopoletin (5280460), Theanine (439378), Tranilast (5282230), Vacuolin-1 (9661141). More details about the structure and functions of representative MC stabilizers can be found elsewhere [178].

Two different pharmacological approaches, not necessarily mutually exclusive, could be followed to address and inhibit MC activity in pathophysiological situations like liver fibrosis: i) MC stabilizers, such as disodium cromoglycate (cromolyn sodium), Tranilast, ketotifen, or many others (Figure 4); and/or ii) inhibitors of MC-selective enzymes (e.g., tryptase and chymase), and antagonists for receptors of typical MC mediators, such as histamine (receptors). These mediators importantly add to the pleiotropic inflammatory functions of MCs (cf. Figure 2).

Already in 1972, results from a controlled study of cromolyn sodium in patients with asthma came to the conclusion that this drug is a useful adjunct to the pharmacological treatment of asthma [179]. Since it was shown in rat peritoneal MCs that cromolyn sodium is able to attenuate IgE-mediated histamine release [180], MCs were seen as the cellular targets of this drug, fitting to their well-known detrimental role in anaphylaxis and asthma. In addition to using cromolyn sodium in patients and experimental rat models, researchers were increasingly using this drug in mouse models to prove the contributions of MCs. However, in a previous study by Galli and coworkers, in which MC-related effects of cromolyn sodium in vitro and in vivo were thoroughly compared in rat and mouse models, the authors came to the conclusion that cromolyn sodium’s effectiveness and selectivity as a MC stabilizer in mice is questionable [159,181]. Nevertheless, in an in vitro model, they could demonstrate the positive effect of histamine secreted from MCs on the proliferation and activation of cholangiocytes, as well as HSCs [181]. In a model of bleomycin-induced pulmonary fibrosis, another MC stabilizer, Tranilast, was shown to be effective in suppressing fibrosis in genetically MC-deficient mice [182], raising doubt about the MC selectivity of this drug, at least in mice.

Nowadays, there are many reports testing mast cell stabilizers, such as luteolin and curcumin in vivo for their efficacy at protecting against inflammation, disease-associated apoptosis, tumorigenesis, and overshooting oxidative stress in the liver [183,184,185,186,187]. Unfortunately, in all these studies, the impact of these stabilizers on mast cell activity was not analyzed systematically. With respect to the mitigation of liver fibrosis, convincing studies have been published with respect to the role of MC proteases, tryptase and chymase. Tryptase β forms non-covalent ring-like tetramers and has trypsin-like activity [188,189]. Tetramerization appears to be required for proper activity and selectivity. The central pore containing the active sites of the four tryptase β molecules prohibits the access of large substrates and also suppresses inhibition by most protease inhibitors [190]. In a model of BDL-induced hepatic fibrosis in rats, treatment with the tryptase inhibitor APC366 resulted in a reduced hepatic fibrosis score, attenuated HSC proliferation, collagen content, and serum biochemical parameters [146]. In contrast to tryptase β, tryptase α is proteolytically inactive; nevertheless, it is conserved throughout evolution and significantly expressed in MCs. Very recently, Le et al. have reported the in vivo existence of biologically functional α/β tryptase heterotetramers; however, their contribution to fibrotic diseases has not been investigated yet [191].

In addition to certain soluble substrates, the G-protein-coupled receptor PAR-2 is cleaved by tryptase and appears to be its main substrate [192]. The tryptase-mediated, N-terminal cleavage of PAR-2 generates an intramolecular ligand activating PAR-2. PAR-2 has been shown to be expressed, amongst others, on fibroblasts and HSCs, and thus, in addition to tryptase inhibitors, PAR-2 antagonists might be beneficial in the treatment of liver fibrosis. Concerning liver fibrosis, rat HSCs express PAR-2 and the expression increases with the transition of stellate cells to myofibroblasts. The PAR-2 agonists, tryptase and the peptide SLIGRL, induced proliferation and collagen secretion [145], suggesting that tryptase as a PAR-2 agonist could sustain liver fibrosis. Indeed, analyzing CCl_4_-induced liver fibrosis in PAR-2-deficient compared to wild-type mice, Knight et al. found a reduced progression of liver fibrosis, hepatic collagen gene expression, hydroxyproline content, TGF-β expression, and matrix metalloproteinase 2 gene expression in the absence of PAR-2. Additionally, PAR-2 activation stimulated proliferation, collagen production, and TGF-β production by human HSCs [144]. This indicated the pro-fibrogenic action of hepatic PAR-2 activation and strongly proposed a role for hepatic MCs in liver fibrosis. Interestingly, the recently identified α/β tryptase heterotetramer was also shown to cleave and activate PAR-2 [191].

Pepducins are cell-penetrating peptides acting as intracellular modulators of signal propagation from receptors of the G-protein-coupled receptor family to the associated G proteins [193]. Intriguingly, the PAR-2 pepducin PZ-235 significantly suppressed hepatic steatosis and inflammation in the experimental methionine-choline-deficient diet model in mice [194]. Moreover, PZ-235 repressed CCl_4_-induced liver fibrosis, even with delayed treatment. PZ-235 also inhibited production of reactive oxygen species and hence, enhanced viability of hepatocytes in vitro [194], which might imply a reduction of necrosis-driven sterile inflammation in the course of liver fibrosis. Thus, PAR-2 pepducin inhibitors have the potential to be efficient in the treatment of liver fibrosis.

Whereas tryptase forms heparin-stabilized ring-like tetramers, which restrict the access of substrates to the active centers of the protease monomers, chymase is active as a monomeric protein, though in a macromolecular complex with heparin [195]. Accordingly, its structure sterically allows for activity against a bigger number of substrates. For instance, chymase can generate angiotensin II by cleaving the non-bioactive peptide angiotensin I [148], and cleave/activate MMP-9 [196] and TGF-β [197], all of which are associated with liver inflammation and/or fibrosis. Furthermore, human chymase was found to cleave type I pro-collagen and hence initiate collagen fibril formation [152]. Isolated rat HSCs were treated with chymase and it was shown that the proliferation and expression of α-smooth muscle actin and TGF-β1 protein were significantly enhanced in a dose-dependent manner. This implied a potential role of chymase in the development of liver fibrosis [151]. Moreover, the effect of the chymase inhibitor TY-51469 in hamsters fed a methionine- and choline-deficient (MCD) diet, which developed a marked hepatic steatosis and fibrosis, was analyzed. Both non-alcoholic steatohepatitis (NASH) and fibrosis were significantly attenuated by chymase inhibition [198,199]. Likewise, in rats fed a high-fat and high-cholesterol diet, the chymase inhibitor TY-51469 significantly attenuated all parameters associated with NASH and fibrosis [200]. Furthermore, CCl_4_-induced liver fibrosis in hamsters was significantly reduced in TY-51469-treated compared to placebo-treated animals [201]. Finally, immunohistochemical analysis, as well as enzymatic activity measurements, of liver biopsies from 49 patients with chronic hepatitis revealed increased chymase levels and activity correlating with the severity of the disease. This suggested that hepatic chymase is implicated in liver fibrosis [202]. A correlative investigation of 77 patients with the aim to elucidate the function of chymase as an angiotensin-converting enzyme for the progression of liver fibrosis suggested an important role of this MC protease in the hepatic fibrosis of patients with cirrhosis [147].

### 5.2. Signaling Pathways as Targets for Potential Future Therapies in Liver Fibrosis

Another critical inflammatory mediator within the fibrotic process in the liver is the cytokine IL-1β [203]. IL-1β is a peculiar cytokine in that it is not translated by ER membrane-bound ribosomes and not secreted via the classical ER–Golgi route [204]. In contrast, pro-IL-1β is synthesized by cytoplasmic ribosomes, and for maturation and secretion, the inflammasome, a multiprotein complex (e.g., the NLRP3 inflammasome), is required for caspase-1 activation and the subsequent cleavage of pro-IL-1β to yield mature IL-1β, which then is released by the process of pyroptosis [204]. Human chymase has been demonstrated to convert the 31 kDa pro-form of IL-1β to an 18 kDa biologically active molecule [205]. This cleavage product differs from the caspase-1-processed molecule in three amino acids; however, the activity of both cytokines is comparable. Since pro-IL-1β can be released, amongst others, from necrotic cells in an alarmin-like fashion, MC chymase could contribute to the generation of IL-1β and hence to the promotion of fibrosis.

So far, the described functions of the MC proteases, tryptase and chymase, for the development of liver fibrosis and associated hepatic alterations indicated a promoting role for these diseases. However, chymase was also reported to degrade two alarmins, IL-33 and HMGB1 [206], which have been shown to play detrimental pro-fibrotic roles in liver disease. An overproduction of the mRNAs for IL-33 and its receptor ST2 was observed in mouse and human fibrotic livers, and activated HSCs were identified as the major source of IL-33 [207]. IL-33 expression in liver was sufficient for severe hepatic fibrosis in vivo by the activation and expansion of liver-resident innate lymphoid cells (ILC2), which then contribute to fibrosis by producing IL-13 [208,209,210]. With respect to the second alarmin, HMGB1, which is produced and released by hepatocytes and Kupffer cells, and signals via the receptor for advanced glycation end-products (RAGE) in HSCs, activation of MAPK pathways and the induction of the increased deposition of collagen has been described [211,212]. In conclusion, chymase is a double-edged sword with regard to the development of liver fibrosis; on the one hand, it cleaves/activates pro-fibrotic proteins, such as angiotensin I, MMP-9, TGF-β, and type I pro-collagen, and on the other hand, it can degrade pro-fibrotic IL-33 and HMGB1. Thus, a thorough analysis of the respective disease development and regulators involved has to be performed before chymase inhibition can be recommended.

Another central MC mediator is the biogenic amine, histamine, which also has been reported to be involved in the regulation of fibrotic diseases. Histamine is synthesized by decarboxylation of the amino acid histidine via the enzyme L-histidine decarboxylase (HDC) [213]. Degradation of histamine is catalyzed by the enzyme monoamine oxidase B (MAOB) [213]. As with tryptase and chymase, histamine as a preformed mediator is stored in secretory lysosomes and is released by the process of degranulation. The effect(s) of histamine on target cells is dependent on the type of histamine receptor(s) expressed by these cells. Histamine receptors (H1R, H2R, H3R, and H4R) belong to the family of G-protein-coupled receptors and exert their differential functions by coupling to different G proteins, which again regulate differential signaling enzymes/pathways. Whereas H1R via G_q_ proteins activates PLC-γ, and thus induces Ca^2+^- and PKC-regulated signaling enzymes and transcription factors, H2R via G_s_ proteins positively couples to adenylyl cyclase, and thus regulates cellular processes by means of cAMP/PKA-dependent signal transduction and gene transcription. In an opposite fashion, H3R and H4R via G_i_ proteins inhibit adenylyl cyclase [214]. Thus, although histamine is only a single substance, it can trigger multiple cellular reactions and affect diseases in various ways.

In the livers of infants suffering from biliary atresia, histamine levels were significantly increased and positively correlated with the severity of fibrosis. Infants with severe fibrosis showed an elevated and reduced expression of HDC and DIO, respectively [213]. The *Abcb4* gene (aka *Mdr2*) encodes for the multidrug-resistant protein MDR2, and *Mdr2*^−/−^ mice spontaneously develop severe biliary fibrosis via gross dysregulation of pro- and anti-fibrotic genes [215]. Interestingly, using H1R and H2R antagonists (mepyramine and ranitidine, respectively) alone or in combination, liver and biliary damage, as well as fibrosis in *Mdr2*^−/−^ mice, was attenuated [160]. Moreover, these H1R and H2R antagonists also decreased the growth of CCA, angiogenesis, and epithelial–mesenchymal transition [160], clearly demonstrating the detrimental role for histamine in liver pathology.

Though so far not studied in liver, histamine was shown to affect the biology of fibroblasts in several ways. Stimulation of normal adult human lung fibroblasts with histamine enhanced the proliferation of these cells in a dose-dependent manner, mediated through the histamine receptor H2R and not H1R, which indicated a role for cAMP/PKA signaling [216]. These data were obtained using the H2R antagonist cimetidine and the H1R antagonist pyrilamine maleate. In normal human dermal fibroblasts, histamine treatment caused a clear enhancement in α-SMA expression, suggesting a role for histamine in fibroblast–myofibroblast progression [217]. Moreover, in human skin fibroblasts, histamine was found to increase proliferation and collagen production [218]. The action of histamine; however, is not only dependent on the histamine receptor(s) expressed, but also on the microenvironment of the respective cells. Hence, Lin et al. demonstrated that TGF-β1-induced expression of α-SMA in human skin fibroblasts was suppressed by histamine. In this situation, H1R activation, but not H2R or H4R activation, was responsible for the suppressive effect by histamine [219]. These few examples already clearly indicate that thorough knowledge about histamine receptor expressing cells in tissues of interest, and in addition, about the type(s) of histamine receptors expressed in these cells, must be obtained to take advantage of the substantial tool-box of various histamine receptor antagonists.

## 6. Conclusions

MCs are important immune cells of the myeloid lineage present in healthy liver, and to a larger degree in diseased liver. In particular, experimental models of hepatic fibrosis have shown that the overall quantities of MCs significantly increase in all kinds of liver insults. In humans, first evidence has connected elevated numbers of MCs to the pathogenesis of PBC, PSC, bile duct obstruction, hepatitis, alcohol-induced liver injury, steatosis, steatohepatitis, congenital and non-congenital liver fibrosis, liver cancer, liver rejection upon liver transplant, and liver aging. Although the impact of MCs in the initiation or progression of these disease entities is still poorly understood, it was suggested that they contribute to the liver’s fibrotic response to chronic inflammation. However, MCs possess favorable immunomodulatory properties on other immune cells and act as a first effector cell in the innate response when encountering antigens. In this regard, the role of MCs in hepatic fibrosis is unclear and merits particular interest. Potential therapeutic MC-targeted strategies are the blockade of MC activity by MC stabilizers, inhibition of MC-selective enzymes, and the application of antagonists binding to receptors associated with an MC function. However, none of these approaches has been systematically tested in appropriate models and further experimental, translational, and clinical studies are urgently needed to explore whether the different compounds are beneficial in the therapy of hepatic fibrosis or its progression to cirrhosis or hepatocellular carcinoma.

## Figures and Tables

**Table 1:** Established mouse injury models that have been the focus of liver-related mast cell research.

Model	Procedure and Typical Time of Sacrifice	Outcome	Model for Human Liver Disease	Reference
CCl_4_	Single or repeated i.p. application (1 h–6 wks), regular inhalation or application by gavage (11–15 wks)	Early: inflammation; late: centrilobular liver damage, fibrosis/cirrhosis induced by forming radicals	Intoxication, acute liver damage, fibrosis	[75]
LPS LPS/D-GalN LPS/BCG	I.p. injection or application of LPS in drinking water alone or in combination with D-GalN; i.p. injection of LPS/BCG (2–8 h)	Inflammation (LPS), acute hepatic failure (LPS/D-GalN), lethal hepatitis (LPS/BCG)	Acute systemic and hepatic inflammation; lethal hepatitis	[79]
DEN DMN	I.p., oral (drinking water, diet, gavage), inhalation, intratracheal, or intragastric instillation (6–50 wks)	Time-dependent liver damage (neutrophilic infiltration, extensive centrilobular hemorrhagic necrosis, bile duct proliferation, fibrosis, and bridging necrosis ending in hepatocarcinogenesis)	Early: intoxication, late: fibrosis, cirrhosis, HCC	[81]
BDL	Surgical ligation of the common biliary duct (5 days–4 wks)	Early: liver cell injury, severe inflammation; late: advanced hepatic fibrosis	Early: liver injury and jaundice, late: cholestatic liver diseases	[82,83]
*Mdr2* ^−/−^	Homozygous disruption of the multidrug resistance 2 gene	Significant increase of bilirubin, alkaline phosphatase, aspartate aminotransferase already at age of 6–14 wks	Cholestatic liver injury	[85]

Abbreviations used are: BCG: bacillus Calmette–Guérin, BDL: bile duct ligation, CCl_4_: carbon tetrachloride D-GalN: D-galactosamine, DEN: diethylnitrosamine, DMN: dimethylnitrosamine, h: hour(s), HCC: hepatocellular carcinoma; i.p.: intraperitoneal, LPS: lipopolysaccharide, wks: weeks.
